# An Update on the Immunotherapy for Oropharyngeal Squamous Cell Carcinoma

**DOI:** 10.3389/fonc.2022.800315

**Published:** 2022-03-15

**Authors:** Yaxuan Huang, Yunyun Lan, Zhe Zhang, Xue Xiao, Tingting Huang

**Affiliations:** ^1^Department of Otorhinolaryngology and Head and Neck Surgery, First Affiliated Hospital of Guangxi Medical University, Nanning, China; ^2^Department of Radiation Oncology, First Affiliated Hospital of Guangxi Medical University, Nanning, China; ^3^Institute of Environmental Medicine, Karolinska Institutet, Stockholm, Sweden

**Keywords:** oropharyngeal squamous cell carcinoma (OPSCC), immunotherapy, immune checkpoint inhibitor, human papillomavirus infection, treatment outcome

## Abstract

Oropharyngeal squamous cell carcinoma (OPSCC) is an uncommon malignancy worldwide. Remarkably, the rising incidence of OPSCC has been observed in many developed countries over the past few decades. On top of tobacco smoking and alcohol consumption, human papillomavirus (HPV) infection has become a major etiologic factor for OPSCC. The radiotherapy-based or surgery-based systemic therapies are recommended equally as first-line treatment, while chemotherapy-based strategy is applied to advanced diseases. Immunotherapy in head and neck squamous cell carcinoma (HNSCC) is currently under the spotlight, especially for patients with advanced diseases. Numerous researches on programmed death-1/programmed death-ligand 1 checkpoint inhibitors have proven beneficial to patients with metastatic HNSCC. In 2016, nivolumab and pembrolizumab were approved as the second-line treatment for advanced metastatic HNSCC by the USA Food and Drug Administration. Soon after, in 2019, the USA Food and Drug Administration approved pembrolizumab as the first-line treatment for patients with unresectable, recurrent, and metastatic HNSCC. It has been reported that HPV-positive HNSCC patients were associated with increased programmed death-ligand 1 expression; however, whether HPV status indicates different treatment outcomes among HNSCC patients treated with immunotherapy has contradicted. Notably, HPV-positive OPSCC exhibits a significantly better clinical response to primary treatment (i.e., radiotherapy, surgery, and chemotherapy) and a more desirable prognosis compared to the HPV-negative OPSCC. This review summarizes the current publications on immunotherapy in HNSCC/OPSCC patients and discusses the impact of HPV infection in immunotherapeutic efficacy, providing an update on the immune landscape and future perspectives in OPSCC.

## 1 Introduction

Oropharyngeal squamous cell carcinoma (OPSCC) is one of the head and neck squamous cell carcinoma (HNSCC), developing in the following areas: soft palate, base of the tongue, palatoglossal folds, palatine tonsils, valleculae, and posterior pharyngeal wall (1). According to the latest GLOBOCAN estimates, OPSCC is uncommon globally, with an estimated 98,412 new cases (0.5% of all cancers combined) and 48,143 deaths (0.5% of all cancers combined) in 2020 (2). Historically, tobacco smoking and alcohol consumption are considered the most common risk factors for HNSCC, including OPSCC (3, 4). Benefits from successfully controlling tobacco and alcohol use in the western world since a few decades ago, incident OPSCCs related to smoking and drinking have been declining (5–8). However, the overall incidence of OPSCC is still on the rise, along with an increasing subset of HPV-positive cases (5–7, 9).

The standard of care (SoC) for OPSCC, which includes surgery, radiotherapy, and chemotherapy, has continuously improved. However, the prognosis of OPSCC patients remains poor due to late diagnosis, high rates of primary-site recurrence, and lymphatic metastasis (10, 11). Recently, the use of immunotherapy in patients with HNSCC, including OPSCC, has become a hot spot (12–15). Among numerous immunomodulatory agents, programmed death-1 (PD-1)/programmed death-ligand 1 (PD-L1) checkpoint inhibitors have been proven effective in those patients with metastatic HNSCC (16–18). Nevertheless, the influence of the HPV status in OPSCC patients on the efficacy of immunotherapy, drug resistance, and heterogeneity on response remain unclear, which are under investigation (19, 20).

This review intends to update the current evidence in immunotherapy among OPSCC patients and the impact of HPV infection on the treatment efficacy of immunotherapy, providing future perspectives in OPSCC treatment.

## 2 HPV and OPSCC

HPV is a small non-enveloped, circular, double-stranded DNA virus with epithelial tropism and commonly transmits by sexual contact. It has been reported that HPV infection was attributed to around 20% ~ 60% of OPSCC worldwide (9, 21). Among 200 identified genotypes of HPVs, genotype HPV-16 accounts for over 80% of HPV-positive OPSCC, followed by HPV-18, 31, 33, 35, which are well-known high-risk HPVs (9, 22). E6 and E7, two early viral proteins expressed by high-risk HPV, are mainly involved in developing and maintaining the transformed phenotype of HPV-induced cancers (23). Specifically, the oncoprotein E6 degrades the tumor suppressor p53 and helps escape cell death (21, 24). E7 binds to the retinoblastoma proteins (pRb), promoting the E2F/pRb complex dissociation and releasing E2F, which stimulates the cell re-entering S-phase, leading to escape from oncogene-induce senescence (21, 25). Besides, E6, E7, and E6/E7 contribute to the maintenance of cancer phenotype, epigenetic regulation, microRNAs, DNA damage response, genetic instability, angiogenesis, immune system modulation, telomerase activity *via* a variety of molecules/pathways (25). Although playing a less crucial role, other early proteins, including E1, E2, E4, and E5, participate in completing the viral cell cycle (9, 21).

HPV-positive OPSCC represents distinct prognostic characteristics and genomic patterns compared to HPV-negative disease. Numerous studies have revealed that HPV-positive OPSCCs exhibited better disease-free survival after primary treatment (26–30). A large-scale retrospective analysis has confirmed the prognostic value of HPV status with a remarkable result: HPV-positive OPSCC patients (63.8%, 206 in 323) represented a better 3-year rates of overall survival (82.4%, vs. 57.1% in HPV-negative OPSCC patients; *P*<0.001) (31). Besides, the comprehensive genomic landscape in HPV-positive HNSCC is remarkably different from smoking-related HNSCC (32–34). In general, HPV-positive HNSCCs exhibited a relatively low mutational burden (2.28 mutations per Mb vs. 4.83 mutations per Mb in HPV-negative cases) (32), a high proliferative index, a frequent alteration in the *PIK3CA* pathway, compared to HPV-negative HNSCCs (35). A current study reported that the most frequent mutation exhibited in an OPSCC cohort with 948 subjects was *TP53* (33%), followed by *PIK3CA* (17%) and *KMT2D* (10.6%); and *TP53* was more commonly mutated in the HPV-negative group (mutation rate: 49% vs. 10%, *P* < 0.0005) (36).

The 7^th^ edition of the Union for International Cancer Control and American Joint Committee on Cancer (UICC/AJCC) staging system failed to differentiate the impact of HPV infection on survival (hazard consistency) between stages and lost the capability in predicting features of any stage (37). Hence, the latest released 8^th^ edition has introduced apparent modifications in a new staging algorithm to categorize OPSCC patients into two different systems regarding HPV status. We summarize the difference between the 7^th^ and 8^th^ edition of UICC/AJCC staging systems of OPSCC in the **Supplementary Table**. The College of American Pathologist Guidelines recommended that p16 expression tested by immunohistochemistry is a feasible and reliable surrogate marker for diagnosing HPV-positive OPSCC (38, 39). It is worth noting that discordance between p16 staining and HPV status (e.g., patients with p16^INK4a^-positive/HPV DNA-negative) has been observed and related to differentiated survival (40, 41). Therefore, we shall be aware that p16^INK4a^ alone may not be the best biomarker for prognosis prediction. Alternative and/or complementary biomarker, such as DNA and RNA *in situ* hybridization and other molecular HPV tests, is urgently needed (37, 42).

## 3 Immunotherapy

A significant breakthrough has been achieved in cancer immunotherapy, making it an important weapon in fighting cancer (43–46). According to the Cancer-Immunity Cycle proposed by Chen et al., cancer cells can be effectively eradicated by the immune cells *via* a stepwise process which starts with a successful initiation of cancer immune recognition and accumulation of adaptive immune responses, to cancer cell elimination eventually (47). Nevertheless, the Cancer-Immunity Cycle does not always work desirably in cancer patients; for instance, T-cell-mediated attack might fail to activate due to the suppression by some factors in the tumor microenvironment (48). Each step in the Cycle acts as a potential strategy for cancer immune escape and an eligible target for treatment (49). Among those steps, PD-1/PD-L1 checkpoint axis is most widely studied, which prevents the over-activation of T cells from damaging normal tissues and leads to the potential of tumor immune escape (12). In the past decade, blocking the PD-1/PD-L1 axis by monoclonal antibodies to overcome the immune suppressive signals in cancer patients and promote the reactivation of antitumor response has been well-established as an efficient treatment in a broad range of cancer types (including but not limited to lung cancer, breast cancer, head and neck cancer, pancreatic cancer, and prostate cancer) (50–54).

## 4 Immunotherapy in HNSCC/OPSCC

### 4.1 Clinical Application of Immunotherapy in HNSCC/OPSCC

#### 4.1.1 PD-1/PD-L1 in HNSCC/OPSCC

In the past decade, dozens of clinical trials have demonstrated the superiority of immunotherapy over chemotherapy in prolonging patients’ survival with advanced HNSCC, including patients with OPSCC. Recent clinical practice in immunotherapy is summarized in **Table 1**.

**Table 1:** Clinical practice in immunotherapy among OPSCC patients.

Immunotherapy	Effect	Drug	Ref.
Monoclonal antibody (mAbs)	Targeting molecular involving in tumor-genesis		(55)
Tumor antigen–targeted mAbs	EGFR antagonist	Cetuximab	
Immune checkpoint–targeted mAbs	CTLA4	Ipilimumab and Tremelimumab	
	PD-1	Pembrolizumab and Nivolumab	
	PD-L1	Darvalumab	
Tumor vaccines	Activating tumor-antigen presentation by APC to T cells	vaccinia-based E6/E7 vaccines	
Immune system modulators	Enhancing immune cell activation and expansion	IL-2, IL-1β, IL-6, IL-8, IFN-γ, TNF-α, G-CSF, GM-CSF, IRX-2, etc.	(56–58)
Stimulatory receptor agonists	Enhancing positive co-stimulatory pathways, providing cytokines	Agonists for CD40	(59)
		Agonists for toll-like receptor	
T-cell transfer therapy	Transfer of *ex vivo* expanded/modulated tumor-reactive T cells into patients.	Tumor-infiltrating lymphocytes (TIL) therapy, Chimeric antigen receptor(CAR) T-cell therapy	(60, 61)

mAbs, Monoclonal antibody; EGFR, epidermal growth factor receptor; CTLA4, Cytotoxic T-Lymphocyte Associated Protein 4; APC, antigen-presenting cell (62–69).

Landmark trials have demonstrated the efficacy of immunotherapy in patients with HNC (including OPSCC). The phase Ib trial published in 2016, KEYNOTE-012 (NCT01848834), was the first study investigating PD-1 blockade therapy in 104 recurrent/metastasis (R/M) HNSCC patients expressing PD-L1 (38% were HPV-positive and 62% were HPV-negative) (70). The overall response rate (ORR) reached 18% (95% CI, 8-32%), and median overall survival (OS) was 13 months. CheckMate 141, a phase III trial, revealed that nivolumab was superior to standard, single-agent therapy (cetuximab, methotrexate, or docetaxel) among 361 patients with platinum-refractory HNSCC (p16-positive/negative were 25.5% and 23.8%, respectively) (43–45). The response rate (RR) in the nivolumab group was 13.3% (95% CI, 9.3 to 18.3) versus 5.8% (95% CI, 2.4 to 11.6) in the standard group; and the OS was significantly longer in the nivolumab group than the standard, single-agent group (hazard ratio for death, 0.70; 97.73% CI, 0.51 to 0.96; *P*=0.01). Based on these two landmark trials, the FDA approved pembrolizumab and nivolumab as the second-line treatment for R/M HNSCC in 2016. Soon after, the phase II, single-arm study, KEYNOTE-055, demonstrated that 16% (95% CI, 11% to 23%) of the 171 R/M HNSCC patients (22% were HPV+ and 77% were HPV-) refractory to platinum and cetuximab who received pembrolizumab achieved confirmed response, with 8 months median duration of response (range, 2+ to 12+ months) (43–45). Around 64% of all patients treated with pembrolizumab experienced different levels of treatment-related adverse events (trAEs) which was deemed acceptable safety. KEYNOTE-040 (NCT02358031) was a globally randomized, phase III study involving 495 R/M HNSCC patients after platinum-based chemotherapy (24.0% were HPV+ and 76.0% were HPV-) (43–45). In 2019, it proved the superiority of pembrolizumab to chemotherapy in the R/M HNSCC patients [median OS: 8.4 months (95% CI 6.4-9.4) in the pembrolizumab group versus 6.9 months (5.9-8.0) in the standard-of-care (SoC) group, the HR was 0.80 (0.65-0.98)]. In addition, fewer patients treated with pembrolizumab exhibited severe trAEs (grade 3 or worse) [33 (13%) of 246 vs 85 (36%) of 234 in standard-of-care group]. Meanwhile, KEYNOTE-048, a randomized, phase III study, stated that pembrolizumab with chemotherapy (platinum and 5-fluorouracil) was effective and safe as a first-line treatment for R/M HNSCC patients (43% were HPV+), and pembrolizumab alone was an appropriate first-line therapy for PD-L1 positive patients (71). Patients treated with pembrolizumab with chemotherapy had better overall survival than those exposed to cetuximab with chemotherapy in the total population [13.0 months vs 10.7 months, HR 0.77 (95% CI 0.63 - 0.93), *P*=0.0034]. Hence, the FDA had taken one big step forward to approve pembrolizumab as the first-line treatment for patients with R/M HNC in 2019 (43–45).

#### 4.1.2 Immunotherapy-Combined Treatments in HNSCC/OPSCC

SoC of HNSCC, including surgery, radiotherapy, chemotherapy, and targeted therapy, have been well-studied and widely applied in clinics with a proven impact (72). Nevertheless, the efficacy of SoC has reached a plateau, and a novel therapeutic modality is urgently needed. After the notable success of PD-1/PD-L1 achieved in treating advanced HNSCC, researchers have devoted their passion to explore the potential of immunotherapy-combined treatments.

Lenvatinib is a tyrosine kinase inhibitor of several VEGF receptors and could modulate immune suppression in the tumor micro-environment by angiogenesis inhibition. The effectiveness of pembrolizumab in combination with lenvatinib in patients with HNSCC has been supported by a phase II trial (NCT02501096) (72) towards 137 patients with various advanced solid tumors (22 patients suffered HNSCC). The ORR at week 24 at the recommended dose (lenvatinib 20 mg/d, pembrolizumab 200 mg every 3 weeks) of HNSCC patients was 36% (8/22; 95% CI, 17.2% to 59.3%). Large-scale studies are needed to evaluate the long-term safety and efficacy of this combination.

Besides, the impact of ICI therapy combined with chemotherapy was investigated in a wide range of solid tumors. Some of these trials (KEYNOTE-189, 355, 361, 407, 590, 826) observe statistically significant survival benefits (longer PFS, higher estimated rate of overall survival at 12 months) in patients with corresponding cancers (73–78), suggesting adding pembrolizumab to standard chemotherapy for cancer treatment. As described above, the KEYNOTE-048 trial reported that pembrolizumab+chemotherapy improved HNSCC patients’ OS versus cetuximab with chemotherapy (13.0 vs. 10.7 months, *P*=0.0034), while pembrolizumab monotherapy was non-inferior to cetuximab with chemotherapy (11.6 vs. 10.7 months). When stratifying the patient population with CPS score, both arms show survival benefits compared to cetuximab with chemotherapy. However, 85% of the R/M HNSCC patients in the pembrolizumab with chemotherapy group suffered grade 3 or worse all-cause adverse events (AEs), while 55% of the patients in the pembrolizumab monotherapy group endured AEs. It indicates that when clinicians decide on monotherapy or combined therapy, the toxicity should be considered in the clinical settings.

#### 4.1.3 Dual Immune Checkpoint Blockade Therapy in HNSCC/OPSCC

The desirable effect of single-agent immunotherapy sparks research into the combination of anti-CTLA-4 and anti-PD-1 therapies (79). Ipilimumab, a monoclonal antibody medication targeting CTLA-4, has been approved for application in melanoma, advanced high-risk renal cell carcinoma, colorectal cancer (80–82). A phase II, open-label, randomized clinical trial (NCT02919683) evaluating the effect of nivolumab (N arm) or nivolumab+ipilimumab (N+I arm) in HNSCC patients before surgical resection showed that both arms had a favorable response, whereas RR in the N+I arm was better in the RECIST manner (N+I arm: 38%, N arm: 13%) (83).

In addition, a phase III trial, EAGLE (NCT02369874), aimed to assess the combination of durvalumab plus tremelimumab (anti-CTLA4 mAb, approved by the FDA to treat malignant mesothelioma) (84). This trial investigated the efficacy of durvalumab +/- tremelimumab versus SoC (e.g., cetuximab, taxane, methotrexate, or fluoropyrimidine) towards 736 R/M HNSCC patients (37.2% with primary OPSCC). Neither in durvalumab arm (D arm) nor in durvalumab plus tremelimumab arm (D+T arm) reported significant survival difference when compared to SoC group (OS: D arm vs. SoC arm, P=0.20; D+T arm vs. SoC arm, P=0.76). Further research is needed to investigate dual ICI therapy’s efficacy in HNSCC, especially OPSCC patients (85).

#### 4.1.4 Novel Immunotherapy in HNSCC/OPSCC

Apart from immune-combined therapy and a combination of anti-CTLA4 and anti-PD-1/PD-L1 therapy, there are other immunotherapies toward HNSCCs using anti-PD-L1 drugs (e.g., avelumab and atezolizumab) (86, 87). Besides, other immunotherapies targeting additional immune checkpoints (e.g., LAG-3, TIM-3, TIGIT, and VISTA) are under investigation (88).

Among these novel therapies, researchers in the field of HNSCC, including OPSCC, start to explore the effect of IDO1 inhibition therapy and toll-like receptor 8 (TLR8) agonists therapy through clinical trials. The results of the phase Ib study (NCT02471846) were disappointing that the combination of navoximod (IDO1 inhibitors) and atezolizumab (anti-PD-L1 agent) failed to improve clinical benefit among patients with various solid tumors, including HNSCC (89). However, the results of the Active8 study were encouraging that TLR8 agonists might prolong survival among HPV-positive HNSCC patients compared to HPV-negative patients (PFS: 7.8 versus 5.9 months; HR, 0.58; P = 0.046; OS: 15.2 versus 12.6 months; HR:0.41; *P* = 0.03) (59).

In all, immunotherapy provides a promising future, but the application in the treatment of OPSCC is still lacking. Researchers should take steps to discover more information in this field.

### 4.2 Immunotherapy in OPSCC Regarding the HPV Status

#### 4.2.1 Immunotherapy in HPV-Positive OPSCC

**Figure 1:**
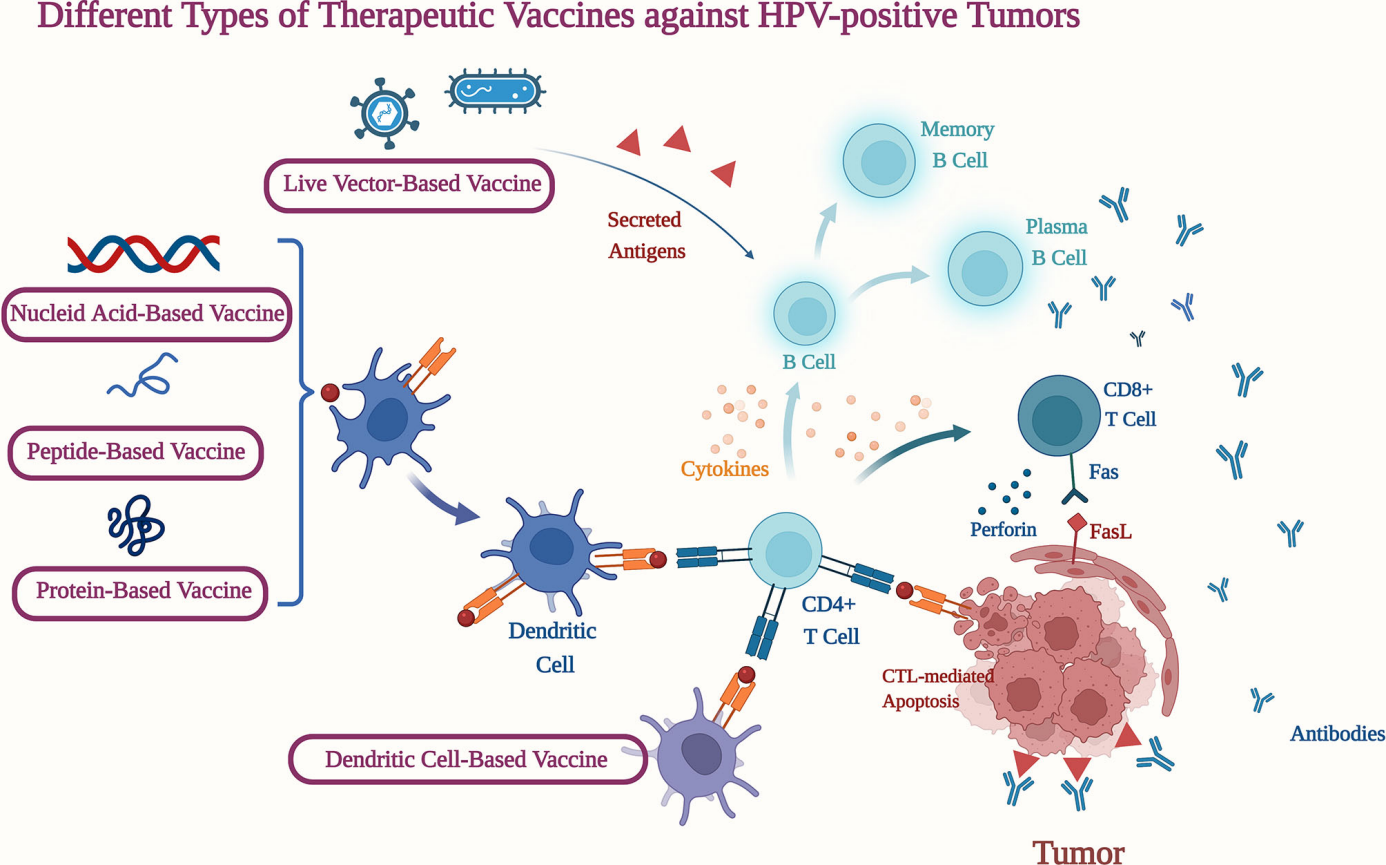
Different Types of Therapeutic Vaccines against HPV-positive Tumors. CD4+ T Cell, Cluster of differentiation 4-positve thymus cell; CD8+ T Cell, Cluster of differentiation 8-positve thymus cell; Fas, Factor associated suicide; FasL, Factor associated suicide ligand. (Figure was created with BioRender.com.)

HPV is a solid causative agent in the formation and progression of OPSCCs, making viral neoantigens an attractive target for therapeutic immunization. Tumor vaccine aims to reduce tumor burden and control tumor recurrence by stimulating both humoral and cellular immune response, offering an immune activation strategy (90). Current HPV-positive therapeutic vaccines are depicted in **Figure 1**.

Given the essential roles E6 and E7 play in HPV-positive cancers, they are usually selected as targets for a therapeutic vaccine. A phase I study (ACTRN12618000140257) assessed the safety, tolerability, and immunogenicity of an HPV E6/E7 vaccine (AMV002) in patients with HPV-positive OPSCC (91). The vaccine-induced RR was 83.3% (10 of 12). In addition, a phase Ib/II trial (NCT03162224) evaluating the safety and efficacy of MEDI0457 (DNA vaccine targeting HPV-16/18 E6/E7 antigens accompanied with an IL-12 adjuvant) plus durvalumab in HPV-positive R/M HNSCC patients is underway (92).

On top of various vaccines, Ramireddy et al. showed that tumor membrane vesicle (TMV) vaccine comprises glycolipid-anchored immuno-stimulatory molecules GPI-B7-1 and GPI-IL-12, magnified the efficacy of anti-PD1 antibodies and inhibited tumor growth, and thus improved the survival of mice with stage VII SCC (93). In addition, the early expressed HPV E5 protein has gained attention recently (94). By amplifying EGFR, HPV E5 protein promoted cell proliferation and invasion through Ras-ERK1/2, PI3K-AKT, and COX-2 pathways (95). As more signaling pathways are involved in understanding tumor-related events, new target sites are under active investigation.

However, those HPV therapeutic vaccines were neither successful in achieving desired clinical outcomes nor applied to human study. Researchers put forward that one reason might be that patients obtained immune tolerance to antigens due to chronic exposure to the virus for years (96). Therefore, we suggest that improving the immunogenicity of several types of vaccines and ensuring safety and tolerability warrant more future efforts.

#### 4.2.2 Immunotherapy in HPV-Negative OPSCC

Currently, there are few studies on immunotherapy towards HPV-negative OPSCC. DURTRERAD is a randomized phase II trial evaluating feasibility and efficacy of durvalumab (D arm) versus durvalumab and tremelimumab (DT arm) in combination with radiotherapy as primary treatment for locally advanced HPV-negative HNSCC, more than a half being OPSCC (97). In this trial, the DT arm was halted due to increased toxicity. Five among six patients in the DT arm suffered varying degrees of trAEs, with one quitting the cohort because of grade 5 trAE. However, detailed analysis has not been fully reported (97).

#### 4.2.3 Survival Benefits of Immunotherapy in Relation to HPV Status

As mentioned above, the prognosis of OPSCC patients is closely associated with HPV status in the setting of SoC. Nevertheless, HPV status seems to be limited in predicting the prognosis of immunotherapies towards OPSCC patients. A systematic review of clinical trials using immunotherapy, mainly ICIs in HNSCC, did not report any statistically significant difference concerning ORR, stable disease (SD), progressive disease (PD), or OS when patients were classified by HPV status. However, ORR was approaching significance in HPV-positive patients compared to HPV-negative ones (21.2% vs. 15.0%, *P* = 0.06) (7).

It should be pointed out that the accuracy of existing detection methods would influence the result of these trials. A study held by Miren Taberna et al. Proves that the ICON-S model in 8th AJCC predicts overall survival assessment better in HPV-related OPC patients when using at least two biomarkers to define HPV-causality (HPV-DNA and (p16INK4a or HPV-mRNA) or double positivity for HPV-DNA/p16INK4a) (98). These results call for a clear definition of HPV status in future studies, and competent biomarkers to identify the beneficiaries from immunotherapies.

### 4.3 Biomarkers for Immunotherapy in OPSCC

Cancer biomarkers help evaluate treatment effects, monitor tumor recurrence and predict survival. The application of biomarkers will influence patient outcomes; hence, exploring and discovering novel biomarkers have significant research value.

The prediction value of PD-(L)1 expression measured by immunohistochemistry for prognosis in patients with HNSCC was analytically validated (99). Previous studies evaluating durvalumab treatment effect demonstrated a better anti-tumor response in R/M HNSCC patients with high PD-L1 expression versus those with low or no PD-L1 expression (median OS: 7.1 vs 6.0 months, ORR: 16.2% vs 9.2%) (100). Nevertheless, the cut-off value varies from trials to trials, and researchers need more trials to define a uniform standard when using PD-(L)1 expression as a prognostic marker (100).

Recent efforts to excavate molecular biomarkers through next-generation sequencing provided deeper and broader insights. Genetic and epidemic alterations are involved in the initiation of HNSCC formation. Genetic alterations include the classic mutations in *TP53* and *CDK2NA* and the newly discovered mutations in *FBXW7*, *TP63*, *IRF6*, and *NOTCH1* (32). NOTCH signaling pathways were associated with the development of multiple types of tumors, such as hepatocellular carcinoma, T cell leukemias, myeloid leukemia (101–103). Gianluigi Grilli et al. reported that the activation of the NOTCH pathway improved prognosis in HPV-negative HNSCC patients and suggested that NOTCH1 expression might be a predictive marker for survival in HPV-negative HNSCC (104). Besides, Esposti et al. identified a unique epigenetic feature: hypomethylation in *NCAN*, *NRXN1*, *COL19A1*, *SYCP2*, *RPA2*, and *SMC1B*, related to HPV infection among HNSCCs regardless of the anatomic site (105). Moreover, differentially expressed small non-coding RNA molecules (miRNAs) may also predict survival. Expression of miR-21 was associated with poor cancer-specific survival in HPV-negative tumors (106).

## 5 Limitations and Prospects

### 5.1 Resistance to Immune Checkpoint Blockade

Most clinical trials evaluating the RR to ICIs in OPSCC patients indicate that less than 15% of patients receiving ICI therapy could achieve durable responses (14, 15, 107). Evidence supports that failure of immune sensing might contribute to compromised immune function. Two dysfunctional oncogenic pathways, the SOX2-mediated suppression of the IFN-I signaling pathway and the PI3K-mTOR pathway, deprive extracellular glucose and thus exhaust cytotoxic T lymphocytes (CTLs) (108). Pervasive immune suppression is the primary barrier, contributing to the limited beneficial effects of ICIs in OPSCC. Researchers proposed that targeting the IFN-I signaling pathway through IFN-I agonists (e.g., cGAMP), inducing DNA damage by RT or DNA-damage inducing agents (e.g., cisplatin and 5-fluorouracil), and is revitalizing CTLs (e.g., rebuilding a pH-neutralized environment to provide nutrition) might be effective (109–112). In addition, Zhou L. et al. suggest that epigenetic targeting drugs such as DNA methyltransferase inhibitors, histone deacetylase, and methyltransferase inhibitors may potentially reverse immune suppression in various cancer models (113).

### 5.2 Difficulty in Personalized Immunotherapy Strategy

In the clinical practice of immunotherapy, evaluating patients’ immune status could be challenging. As discussed above, feasible biomarkers could guide researchers to identify potential beneficial patients and monitor adverse events. However, it is insufficient to select patients for ICI immunotherapy based on a single parameter without considering other factors given the highly heterogeneous microenvironment in OPSCC. An *ex vivo* platform, CANscript system, has been proven helpful to for profiling the response of immunotherapy combining chemotherapy (114). Besides, researchers have established several prognosis risk models to indicate immunosuppression state and predict survival in patients based on a set of immune checkpoint-related genes (115, 116). It is a pity that, however, existing predictive models lack validation upon large sample size.

## 6 Conclusion

In summary, classified by HPV status, OPSCC is a heterogeneous disease. The unique TME shaping by HPV status calls for distinct therapeutic approaches. Immunotherapy offers a wide range of therapeutic strategies which will be especially useful in meeting this need. Monotherapy of novel agents has proved effective, while combinations of immunotherapy with conventional therapies and dual immunotherapy are undergoing clinical investigation. Notably, there is an urgent need for a feasible treatment stratification by HPV status. Immunosuppression and lack of desirable biomarkers for personalized therapy are the two significant issues in immunotherapy. More clinical trials are warranted to assess the efficacy of novel immunotherapies based on HPV status.

## Author Contributions

All authors listed have made a substantial, direct, and intellectual contribution to the work, and approved it for publication.

## Conflict of Interest

The authors declare that the research was conducted in the absence of any commercial or financial relationships that could be construed as a potential conflict of interest.

## Publisher’s Note

All claims expressed in this article are solely those of the authors and do not necessarily represent those of their affiliated organizations, or those of the publisher, the editors and the reviewers. Any product that may be evaluated in this article, or claim that may be made by its manufacturer, is not guaranteed or endorsed by the publisher.

## References

[1] Chi AC Day TA Neville BW. Oral Cavity and Oropharyngeal Squamous Cell Carcinoma–an Update. CA Cancer J Clin (2015) 65(5):401–21. doi: 10.3322/caac.21293 26215712

[2] Sung H Ferlay J Siegel RL Laversanne M Soerjomataram I Jemal A. Global Cancer Statistics 2020: GLOBOCAN Estimates of Incidence and Mortality Worldwide for 36 Cancers in 185 Countries. CA Cancer J Clin (2021) 71(3):209–49. doi: 10.3322/caac.21660 33538338

[3] Rettig EM D’Souza G. Epidemiology of Head and Neck Cancer. Surg Oncol Clin N Am (2015) 24(3):379–96. doi: 10.1016/j.soc.2015.03.001 25979389

[4] Hashibe M Brennan P Benhamou S Castellsague X Chen C Curado MP. Alcohol Drinking in Never Users of Tobacco, Cigarette Smoking in Never Drinkers, and the Risk of Head and Neck Cancer: Pooled Analysis in the International Head and Neck Cancer Epidemiology Consortium. J Natl Cancer Inst (2007) 99(10):777–89. doi: 10.1093/jnci/djk179 17505073

[5] D’Souza G Kreimer AR Viscidi R Pawlita M Fakhry C Koch WM. Case-Control Study of Human Papillomavirus and Oropharyngeal Cancer. N Engl J Med (2007) 356(19):1944–56. doi: 10.1056/NEJMoa065497 17494927

[6] Gillison ML Castellsagué X Chaturvedi A Goodman MT Snijders P Tommasino M. Eurogin Roadmap: Comparative Epidemiology of HPV Infection and Associated Cancers of the Head and Neck and Cervix. Int J Cancer (2014) 134(3):497–507. doi: 10.1002/ijc.28201 23568556

[7] Patel JJ Levy DA Nguyen SA Knochelmann HM Day TA. Impact of PD-L1 Expression and Human Papillomavirus Status in Anti-PD1/PDL1 Immunotherapy for Head and Neck Squamous Cell Carcinoma-Systematic Review and Meta-Analysis. Head Neck (2020) 42(4):774–86. doi: 10.1002/hed.26036 PMC7147243 31762164

[8] van Monsjou HS Balm AJ van den Brekel MM Wreesmann VB. Oropharyngeal Squamous Cell Carcinoma: A Unique Disease on the Rise? Oral Oncol (2010) 46(11):780–5. doi: 10.1016/j.oraloncology.2010.08.011 20920878

[9] Taberna M Mena M Pavón MA Alemany L Gillison ML Mesía R. Human Papillomavirus-Related Oropharyngeal Cancer. Ann Oncol (2017) 28(10):2386–98. doi: 10.1093/annonc/mdx304 28633362

[10] Liebertz DJ Lechner MG Masood R Sinha UK Han J Puri RK. Establishment and Characterization of a Novel Head and Neck Squamous Cell Carcinoma Cell Line USC-Hn1. Head Neck Oncol (2010) 2:5. doi: 10.1186/1758-3284-2-5 20175927 PMC2841166

[11] Warnakulasuriya S. Global Epidemiology of Oral and Oropharyngeal Cancer. Oral Oncol (2009) 45(4-5):309–16. doi: 10.1016/j.oraloncology.2008.06.002 18804401

[12] Carlisle JW Steuer CE Owonikoko TK Saba NF. An Update on the Immune Landscape in Lung and Head and Neck Cancers. CA Cancer J Clin (2020) 70(6):505–17. doi: 10.3322/caac.21630 32841388

[13] Bauml J Seiwert TY Pfister DG Worden F Liu SV Gilbert J. Pembrolizumab for Platinum- and Cetuximab-Refractory Head and Neck Cancer: Results From a Single-Arm, Phase II Study. J Clin Oncol (2017) 35(14):1542–9. doi: 10.1200/JCO.2016.70.1524 PMC5946724 28328302

[14] Ferris RL Blumenschein G Jr. Fayette J Guigay J Colevas AD Licitra L. Nivolumab for Recurrent Squamous-Cell Carcinoma of the Head and Neck. N Engl J Med (2016) 375(19):1856–67. doi: 10.1056/NEJMoa1602252 PMC5564292 27718784

[15] Cohen EEW Soulières D Le Tourneau C Dinis J Licitra L Ahn MJ. Pembrolizumab Versus Methotrexate, Docetaxel, or Cetuximab for Recurrent or Metastatic Head-and-Neck Squamous Cell Carcinoma (KEYNOTE-040): A Randomised, Open-Label, Phase 3 Study. Lancet (2019) 393(10167):156–67. doi: 10.1016/S0140-6736(18)31999-8 30509740

[16] Mehra R Seiwert TY Gupta S Weiss J Gluck I Eder JP. Efficacy and Safety of Pembrolizumab in Recurrent/Metastatic Head and Neck Squamous Cell Carcinoma: Pooled Analyses After Long-Term Follow-Up in KEYNOTE-012. Br J Cancer (2018) 119(2):153–9. doi: 10.1038/s41416-018-0131-9 PMC6048158 29955135

[17] Ferris RL Licitra L Fayette J Even C Blumenschein G Jr. Harrington KJ. Nivolumab in Patients With Recurrent or Metastatic Squamous Cell Carcinoma of the Head and Neck: Efficacy and Safety in CheckMate 141 by Prior Cetuximab Use. Clin Cancer Res (2019) 25(17):5221–30. doi: 10.1158/1078-0432.CCR-18-3944 PMC7721346 31239321

[18] Siu LL Even C Mesía R Remenar E Daste A Delord JP. Safety and Efficacy of Durvalumab With or Without Tremelimumab in Patients With PD-L1-Low/Negative Recurrent or Metastatic HNSCC: The Phase 2 CONDOR Randomized Clinical Trial. JAMA Oncol (2019) 5(2):195–203. doi: 10.1001/jamaoncol.2018.4628 30383184 PMC6439564

[19] Wang HC Yeh TJ Chan LP Hsu CM Cho SF. Exploration of Feasible Immune Biomarkers for Immune Checkpoint Inhibitors in Head and Neck Squamous Cell Carcinoma Treatment in Real World Clinical Practice. Int J Mol Sci (2020) 21(20):7621. doi: 10.3390/ijms21207621 PMC7589088 33076306

[20] Tan S Li D Zhu X. Cancer Immunotherapy: Pros, Cons and Beyond. BioMed Pharmacother (2020) 124:109821. doi: 10.1016/j.biopha.2020.109821 31962285

[21] Dong Z Hu R Du Y Tan L Li L Du J. Immunodiagnosis and Immunotherapeutics Based on Human Papillomavirus for HPV-Induced Cancers. Front Immunol (2020) 11:586796. doi: 10.3389/fimmu.2020.586796 33488587 PMC7820759

[22] Johnson DE Burtness B Leemans CR Lui VWY Bauman JE Grandis JR. Head and Neck Squamous Cell Carcinoma. Nat Rev Dis Primers (2020) 6(1):92. doi: 10.1038/s41572-020-00224-3 33243986 PMC7944998

[23] Hoppe-Seyler K Bossler F Braun JA Herrmann AL Hoppe-Seyler F. The HPV E6/E7 Oncogenes: Key Factors for Viral Carcinogenesis and Therapeutic Targets. Trends Microbiol (2018) 26(2):158–68. doi: 10.1016/j.tim.2017.07.007 28823569

[24] Estêvão D Costa NR Gil da Costa RM Medeiros R. Hallmarks of HPV Carcinogenesis: The Role of E6, E7 and E5 Oncoproteins in Cellular Malignancy. Biochim Biophys Acta Gene Regul Mech (2019) 1862(2):153–62. doi: 10.1016/j.bbagrm.2019.01.001 30707946

[25] Vats A Trejo-Cerro O Thomas M Banks L. Human Papillomavirus E6 and E7: What Remains? Tumour Virus Res (2021) 11:200213. doi: 10.1016/j.tvr.2021.200213 33716206 PMC7972986

[26] Marur S D'Souza G Westra WH Forastiere AA. HPV-Associated Head and Neck Cancer: A Virus-Related Cancer Epidemic. Lancet Oncol (2010) 11(8):781–9. doi: 10.1016/S1470-2045(10)70017-6 PMC5242182 20451455

[27] Licitra L Perrone F Bossi P Suardi S Mariani L Artusi R. High-Risk Human Papillomavirus Affects Prognosis in Patients With Surgically Treated Oropharyngeal Squamous Cell Carcinoma. J Clin Oncol (2006) 24(36):5630–6. doi: 10.1200/JCO.2005.04.6136 17179101

[28] Lindquist D Romanitan M Hammarstedt L Näsman A Dahlstrand H Lindholm J. Human Papillomavirus is a Favourable Prognostic Factor in Tonsillar Cancer and its Oncogenic Role is Supported by the Expression of E6 and E7. Mol Oncol (2007) 1(3):350–5. doi: 10.1016/j.molonc.2007.08.005 PMC5543872 19383307

[29] Lassen P Eriksen JG Hamilton-Dutoit S Tramm T Alsner J Overgaard J. Effect of HPV-Associated P16ink4a Expression on Response to Radiotherapy and Survival in Squamous Cell Carcinoma of the Head and Neck. J Clin Oncol (2009) 27(12):1992–8. doi: 10.1200/JCO.2008.20.2853 19289615

[30] Kumar B Cordell KG Lee JS Prince ME Tran HH Wolf GT. Response to Therapy and Outcomes in Oropharyngeal Cancer are Associated With Biomarkers Including Human Papillomavirus, Epidermal Growth Factor Receptor, Gender, and Smoking. Int J Radiat Oncol Biol Phys (2007) 69(2 Suppl):S109–11. doi: 10.1016/j.ijrobp.2007.05.072 PMC2084353 17848274

[31] Ang KK Harris J Wheeler R Weber R Rosenthal DI Nguyen-Tân PF. Human Papillomavirus and Survival of Patients With Oropharyngeal Cancer. N Engl J Med (2010) 363(1):24–35. doi: 10.1056/NEJMoa0912217 20530316 PMC2943767

[32] Stransky N Egloff AM Tward AD Kostic AD Cibulskis K Sivachenko A. The Mutational Landscape of Head and Neck Squamous Cell Carcinoma. Science (2011) 333(6046):1157–60. doi: 10.1126/science.1208130 PMC3415217 21798893

[33] Agrawal N Frederick MJ Pickering CR Bettegowda C Chang K Li RJ. Exome Sequencing of Head and Neck Squamous Cell Carcinoma Reveals Inactivating Mutations in NOTCH1. Science (2011) 333(6046):1154–7. doi: 10.1126/science.1206923 PMC3162986 21798897

[34] Cancer Genome Atlas Network Comprehensive Genomic Characterization of Head and Neck Squamous Cell Carcinomas. Nature (2015) 517(7536):576–82. doi: 10.1038/nature14129 PMC4311405 25631445

[35] Sastre-Garau X Harlé A. Pathology of HPV-Associated Head and Neck Carcinomas: Recent Data and Perspectives for the Development of Specific Tumor Markers. Front Oncol (2020) 10:528957. doi: 10.3389/fonc.2020.528957 33312940 PMC7701329

[36] Shaikh H McGrath JE Hughes B Xiu J Brodskiy P Sukari A. Genomic and Molecular Profiling of Human Papillomavirus Associated Head and Neck Squamous Cell Carcinoma Treated With Immune Checkpoint Blockade Compared to Survival Outcomes. Cancers (Basel) (2021) 13(24):6309. doi: 10.3390/cancers13246309 34944929 PMC8699559

[37] Lydiatt WM Patel SG O'Sullivan B Brandwein MS Ridge JA Migliacci JC. Head and Neck Cancers-Major Changes in the American Joint Committee on Cancer Eighth Edition Cancer Staging Manual. CA Cancer J Clin (2017) 67(2):122–37. doi: 10.3322/caac.21389 28128848

[38] Lewis JS Jr Beadle B Bishop JA Chernock RD Colasacco C Lacchetti C. Human Papillomavirus Testing in Head and Neck Carcinomas: Guideline From the College of American Pathologists. Arch Pathol Lab Med (2018) 142(5):559–97. doi: 10.5858/arpa.2017-0286-CP 29251996

[39] Fakhry C Lacchetti C Rooper LM Jordan RC Rischin D Sturgis EM. Human Papillomavirus Testing in Head and Neck Carcinomas: ASCO Clinical Practice Guideline Endorsement of the College of American Pathologists Guideline. J Clin Oncol (2018) 36(31):3152–61. doi: 10.1200/JCO.18.00684 30188786

[40] Shinn JR Davis SJ Lang-Kuhs KA Rohde S Wang X Liu P. Oropharyngeal Squamous Cell Carcinoma With Discordant P16 and HPV mRNA Results: Incidence and Characterization in a Large, Contemporary United States Cohort. Am J Surg Pathol (2021) 45(7):951–61. doi: 10.1097/PAS.0000000000001685 PMC8192336 33739785

[41] Rietbergen MM Brakenhoff RH Bloemena E Witte BI Snijders PJ Heideman DA. Human Papillomavirus Detection and Comorbidity: Critical Issues in Selection of Patients With Oropharyngeal Cancer for Treatment De-Escalation Trials. Ann Oncol (2013) 24(11):2740–5. doi: 10.1093/annonc/mdt319 23946330

[42] Jalaly JB Hosseini SM Shafique K Baloch ZW. Current Status of P16 Immunohistochemistry and HPV Testing in Fine Needle Aspiration Specimens of the Head and Neck. Acta Cytol (2020) 64(1-2):30–9. doi: 10.1159/000496158 30783052

[43] Yang Y. Cancer Immunotherapy: Harnessing the Immune System to Battle Cancer. J Clin Invest (2015) 125(9):3335–7. doi: 10.1172/JCI83871 PMC4588312 26325031

[44] Huang PW Chang JW. Immune Checkpoint Inhibitors Win the 2018 Nobel Prize. BioMed J (2019) 42(5):299–306. doi: 10.1016/j.bj.2019.09.002 31783990 PMC6889239

[45] Almangush A Leivo I Mäkitie AA. Biomarkers for Immunotherapy of Oral Squamous Cell Carcinoma: Current Status and Challenges. Front Oncol (2021) 11:616629. doi: 10.3389/fonc.2021.616629 33763354 PMC7982571

[46] He X Xu C. Immune Checkpoint Signaling and Cancer Immunotherapy. Cell Res (2020) 30(8):660–9. doi: 10.1038/s41422-020-0343-4 PMC7395714 32467592

[47] Chen DS Mellman I. Oncology Meets Immunology: The Cancer-Immunity Cycle. Immunity (2013) 39(1):1–10. doi: 10.1016/j.immuni.2013.07.012 23890059

[48] Motz GT Coukos G. Deciphering and Reversing Tumor Immune Suppression. Immunity (2013) 39(1):61–73. doi: 10.1016/j.immuni.2013.07.005 23890064 PMC3782392

[49] Gavrielatou N Doumas S Economopoulou P Foukas PG Psyrri A. Biomarkers for Immunotherapy Response in Head and Neck Cancer. Cancer Treat Rev (2020) 84:101977. doi: 10.1016/j.ctrv.2020.101977 32018128

[50] Hirsch FR Scagliotti GV Mulshine JL Kwon R Curran WJ Jr. Wu YL. Lung Cancer: Current Therapies and New Targeted Treatments. Lancet (2017) 389(10066):299–311. doi: 10.1016/S0140-6736(16)30958-8 27574741

[51] Emens LA. Breast Cancer Immunotherapy: Facts and Hopes. Clin Cancer Res (2018) 24(3):511–20. doi: 10.1158/1078-0432.CCR-16-3001 PMC5796849 28801472

[52] Sim F Leidner R Bell RB. Immunotherapy for Head and Neck Cancer. Oral Maxillofac Surg Clin North Am (2019) 31(1):85–100. doi: 10.1016/j.coms.2018.09.002 30449528

[53] Morrison AH Byrne KT Vonderheide RH. Immunotherapy and Prevention of Pancreatic Cancer. Trends Cancer (2018) 4(6):418–28. doi: 10.1016/j.trecan.2018.04.001 PMC6028935 29860986

[54] Gamat M McNeel DG. Androgen Deprivation and Immunotherapy for the Treatment of Prostate Cancer. Endocr Relat Cancer (2017) 24(12):T297–310. doi: 10.1530/ERC-17-0145 28814451 PMC5669826

[55] Ferris RL. Immunology and Immunotherapy of Head and Neck Cancer. J Clin Oncol (2015) 33(29):3293–304. doi: 10.1200/JCO.2015.61.1509 PMC4586169 26351330

[56] Verastegui E Barrera JL Zinser J Del Rio R Meneses A de la Garza J. A Natural Cytokine Mixture (IRX-2) and Interference With Immune Suppression Induce Immune Mobilization and Regression of Head and Neck Cancer. Int J Immunopharmacol (1997) 19(11-12):619–27. doi: 10.1016/s0192-0561(97)00059-3 9669202

[57] Ralli M Grasso M Gilardi A Ceccanti M Messina MP Tirassa P. The Role of Cytokines in Head and Neck Squamous Cell Carcinoma: A Review. Clin Ter (2020) 171(3):e268–74. doi: 10.7417/CT.2020.2225 32323717

[58] Choudhary MM France TJ Teknos TN Kumar P. Interleukin-6 Role in Head and Neck Squamous Cell Carcinoma Progression. World J Otorhinolaryngol Head Neck Surg (2016) 2(2):90–7. doi: 10.1016/j.wjorl.2016.05.002 PMC5698512 29204553

[59] Ferris RL Saba NF Gitlitz BJ Haddad R Sukari A Neupane P. Effect of Adding Motolimod to Standard Combination Chemotherapy and Cetuximab Treatment of Patients With Squamous Cell Carcinoma of the Head and Neck: The Active8 Randomized Clinical Trial. JAMA Oncol (2018) 4(11):1583–8. doi: 10.1001/jamaoncol.2018.1888 PMC6248084 29931076

[60] Rosewell Shaw A Porter CE Watanabe N Tanoue K Sikora A Gottschalk S. Adenovirotherapy Delivering Cytokine and Checkpoint Inhibitor Augments CAR T Cells Against Metastatic Head and Neck Cancer. Mol Ther (2017) 25(11):2440–51. doi: 10.1016/j.ymthe.2017.09.010 PMC5675597 28974431

[61] Park YP Jin L Bennett KB Wang D Fredenburg KM Tseng JE. CD70 as a Target for Chimeric Antigen Receptor T Cells in Head and Neck Squamous Cell Carcinoma. Oral Oncol (2018) 78:145–50. doi: 10.1016/j.oraloncology.2018.01.024 PMC5836804 29496042

[62] Dadian G Riches PG Henderson DC MacLennan K Lorentzos A Moore J. Immune Changes in Peripheral Blood Resulting From Locally Directed Interleukin-2 Therapy in Squamous Cell Carcinoma of the Head and Neck. Eur J Cancer B Oral Oncol (1993) 29b(1):29–34. doi: 10.1016/0964-1955(93)90007-2 8180573

[63] De Stefani A Valente G Forni G Lerda W Ragona R Cortesina G. Treatment of Oral Cavity and Oropharynx Squamous Cell Carcinoma With Perilymphatic Interleukin-2: Clinical and Pathologic Correlations. J Immunother Emphasis Tumor Immunol (1996) 19(2):125–33. doi: 10.1097/00002371-199603000-00005 8732695

[64] Colnot DR Quak JJ Roos JC van Lingen A Wilhelm AJ van Kamp GJ. Phase I Therapy Study of 186Re-Labeled Chimeric Monoclonal Antibody U36 in Patients With Squamous Cell Carcinoma of the Head and Neck. J Nucl Med (2000) 41(12):1999–2010. 11138685

[65] Colnot DR Ossenkoppele GJ Roos JC Quak JJ de Bree R Börjesson PK. Reinfusion of Unprocessed, Granulocyte Colony-Stimulating Factor-Stimulated Whole Blood Allows Dose Escalation of 186Relabeled Chimeric Monoclonal Antibody U36 Radioimmunotherapy in a Phase I Dose Escalation Study. Clin Cancer Res (2002) 8(11):3401–6. 12429627

[66] Forni G Giovarelli M Jemma C Bosco MC Caretto P Modesti A. Perilymphatic Injections of Cytokines: A New Tool in Active Cancer Immunotherapy. Experimental Rationale and Clinical Findings. Ann Ist Super Sanita (1990) 26(3-4):397–409. 2151108

[67] Freeman SM Franco JL Kenady DE Baltzer L Roth Z Brandwein HJ. A Phase 1 Safety Study of an IRX-2 Regimen in Patients With Squamous Cell Carcinoma of the Head and Neck. Am J Clin Oncol (2011) 34(2):173–8. doi: 10.1097/COC.0b013e3181dbb9d8 20539208

[68] Jensen AD Krauss J Potthoff K Desta A Habl G Mavtratzas A. Phase II Study of Induction Chemotherapy With TPF Followed by Radioimmunotherapy With Cetuximab and Intensity-Modulated Radiotherapy (IMRT) in Combination With a Carbon Ion Boost for Locally Advanced Tumours of the Oro-, Hypopharynx and Larynx–TPF-C-HIT. BMC Cancer (2011) 11:182. doi: 10.1186/1471-2407-11-182 21595970 PMC3118195

[69] Mantovani G Gebbia V Airoldi M Bumma C Contu P Bianchi A. Neo-Adjuvant Chemo-(Immuno-)Therapy of Advanced Squamous-Cell Head and Neck Carcinoma: A Multicenter, Phase III, Randomized Study Comparing Cisplatin + 5-Fluorouracil (5-FU) With Cisplatin + 5-FU + Recombinant Interleukin 2. Cancer Immunol Immunother (1998) 47(3):149–56. doi: 10.1007/s002620050515 PMC11037387 9829840

[70] Seiwert TY Burtness B Mehra R Weiss J Berger R Eder JP. Safety and Clinical Activity of Pembrolizumab for Treatment of Recurrent or Metastatic Squamous Cell Carcinoma of the Head and Neck (KEYNOTE-012): An Open-Label, Multicentre, Phase 1b Trial. Lancet Oncol (2016) 17(7):956–65. doi: 10.1016/S1470-2045(16)30066-3 27247226

[71] Burtness B Harrington KJ Greil R Soulières D Tahara M de Castro G Jr.. Pembrolizumab Alone or With Chemotherapy Versus Cetuximab With Chemotherapy for Recurrent or Metastatic Squamous Cell Carcinoma of the Head and Neck (KEYNOTE-048): A Randomised, Open-Label, Phase 3 Study. Lancet (2019) 394(10212):1915–28. doi: 10.1016/S0140-6736(19)32591-7 31679945

[72] Taylor MH Lee CH Makker V Rasco D Dutcus CE Wu J. Phase IB/II Trial of Lenvatinib Plus Pembrolizumab in Patients With Advanced Renal Cell Carcinoma, Endometrial Cancer, and Other Selected Advanced Solid Tumors. J Clin Oncol (2020) 38(11):1154–63. doi: 10.1200/JCO.19.01598 PMC7145588 31961766

[73] Gandhi L Rodríguez-Abreu D Gadgeel S Esteban E Felip E De Angelis F. Pembrolizumab Plus Chemotherapy in Metastatic Non-Small-Cell Lung Cancer. N Engl J Med (2018) 378(22):2078–92. doi: 10.1056/NEJMoa1801005 29658856

[74] Cortes J Cescon DW Rugo HS Nowecki Z Im SA Yusof MM. Pembrolizumab Plus Chemotherapy Versus Placebo Plus Chemotherapy for Previously Untreated Locally Recurrent Inoperable or Metastatic Triple-Negative Breast Cancer (KEYNOTE-355): A Randomised, Placebo-Controlled, Double-Blind, Phase 3 Clinical Trial. Lancet (2020) 396(10265):1817–28. doi: 10.1016/S0140-6736(20)32531-9 33278935

[75] Powles T Csőszi T Özgüroğlu M Matsubara N Géczi L Cheng SY. Pembrolizumab Alone or Combined With Chemotherapy Versus Chemotherapy as First-Line Therapy for Advanced Urothelial Carcinoma (KEYNOTE-361): A Randomised, Open-Label, Phase 3 Trial. Lancet Oncol (2021) 22(7):931–45. doi: 10.1016/S1470-2045(21)00152-2 34051178

[76] Paz-Ares L Vicente D Tafreshi A Robinson A Soto Parra H Mazières J. A Randomized, Placebo-Controlled Trial of Pembrolizumab Plus Chemotherapy in Patients With Metastatic Squamous NSCLC: Protocol-Specified Final Analysis of KEYNOTE-407. J Thorac Oncol (2020) 15(10):1657–69. doi: 10.1016/j.jtho.2020.06.015 32599071

[77] Sun JM Shen L Shah MA Enzinger P Adenis A Doi T. Pembrolizumab Plus Chemotherapy Versus Chemotherapy Alone for First-Line Treatment of Advanced Oesophageal Cancer (KEYNOTE-590): A Randomised, Placebo-Controlled, Phase 3 Study. Lancet (2021) 398(10302):759–71. doi: 10.1016/S0140-6736(21)01234-4 34454674

[78] Colombo N Dubot C Lorusso D Caceres MV Hasegawa K Shapira-Frommer R. Pembrolizumab for Persistent, Recurrent, or Metastatic Cervical Cancer. N Engl J Med (2021) 385(20):1856–67. doi: 10.1056/NEJMoa2112435 34534429

[79] Bashir B Wilson MA. Novel Immunotherapy Combinations. Curr Oncol Rep (2019) 21(11):96. doi: 10.1007/s11912-019-0851-x 31696332

[80] Ascierto PA Del Vecchio M Mandalá M Gogas H Arance AM Dalle S. Adjuvant Nivolumab Versus Ipilimumab in Resected Stage IIIB-C and Stage IV Melanoma (CheckMate 238): 4-Year Results From a Multicentre, Double-Blind, Randomised, Controlled, Phase 3 Trial. Lancet Oncol (2020) 21(11):1465–77. doi: 10.1016/S1470-2045(20)30494-0 32961119

[81] Motzer RJ Rini BI McDermott DF Arén Frontera O Hammers HJ Carducci MA. Nivolumab Plus Ipilimumab Versus Sunitinib in First-Line Treatment for Advanced Renal Cell Carcinoma: Extended Follow-Up of Efficacy and Safety Results From a Randomised, Controlled, Phase 3 Trial. Lancet Oncol (2019) 20(10):1370–85. doi: 10.1016/S1470-2045(19)30413-9 PMC7497870 31427204

[82] Overman MJ Lonardi S Wong KYM Lenz HJ Gelsomino F Aglietta M. Durable Clinical Benefit With Nivolumab Plus Ipilimumab in DNA Mismatch Repair-Deficient/Microsatellite Instability-High Metastatic Colorectal Cancer. J Clin Oncol (2018) 36(8):773–9. doi: 10.1200/JCO.2017.76.9901 29355075

[83] Schoenfeld JD Hanna GJ Jo VY Rawal B Chen YH Catalano PS. Neoadjuvant Nivolumab or Nivolumab Plus Ipilimumab in Untreated Oral Cavity Squamous Cell Carcinoma: A Phase 2 Open-Label Randomized Clinical Trial. JAMA Oncol (2020) 6(10):1563–70. doi: 10.1001/jamaoncol.2020.2955 PMC7453348 32852531

[84] Ferris RL Haddad R Even C Tahara M Dvorkin M Ciuleanu TE. Durvalumab With or Without Tremelimumab in Patients With Recurrent or Metastatic Head and Neck Squamous Cell Carcinoma: EAGLE, a Randomized, Open-Label Phase III Study. Ann Oncol (2020) 31(7):942–50. doi: 10.1016/j.annonc.2020.04.001 32294530

[85] Antonia SJ Villegas A Daniel D Vicente D Murakami S Hui R. Durvalumab After Chemoradiotherapy in Stage III Non-Small-Cell Lung Cancer. N Engl J Med (2017) 377(20):1919–29. doi: 10.1056/NEJMoa1709937 28885881

[86] Lee NY Ferris RL Psyrri A Haddad RI Tahara M Bourhis J. Avelumab Plus Standard-of-Care Chemoradiotherapy Versus Chemoradiotherapy Alone in Patients With Locally Advanced Squamous Cell Carcinoma of the Head and Neck: A Randomised, Double-Blind, Placebo-Controlled, Multicentre, Phase 3 Trial. Lancet Oncol (2021) 22(4):450–62. doi: 10.1016/S1470-2045(20)30737-3 33794205

[87] Colevas AD Bahleda R Braiteh F Balmanoukian A Brana I Chau NG. Safety and Clinical Activity of Atezolizumab in Head and Neck Cancer: Results From a Phase I Trial. Ann Oncol (2018) 29(11):2247–53. doi: 10.1093/annonc/mdy411 30219915

[88] Qin S Xu L Yi M Yu S Wu K Luo S. Novel Immune Checkpoint Targets: Moving Beyond PD-1 and CTLA-4. Mol Cancer (2019) 18(1):155. doi: 10.1186/s12943-019-1091-2 31690319 PMC6833286

[89] Sailer V Sailer U Bawden EG Zarbl R Wiek C Vogt TJ. DNA Methylation of Indoleamine 2,3-Dioxygenase 1 (IDO1) in Head and Neck Squamous Cell Carcinomas Correlates With IDO1 Expression, HPV Status, Patients’ Survival, Immune Cell Infiltrates, Mutational Load, and Interferon γ Signature. EBioMedicine (2019) 48:341–52. doi: 10.1016/j.ebiom.2019.09.038 PMC6838413 31628024

[90] Sahin U Türeci Ö. Personalized Vaccines for Cancer Immunotherapy. Science (2018) 359(6382):1355–60. doi: 10.1126/science.aar7112 29567706

[91] Chandra J Woo WP Finlayson N Liu HY McGrath M Ladwa R. A Phase 1, Single Centre, Open Label, Escalating Dose Study to Assess the Safety, Tolerability and Immunogenicity of a Therapeutic Human Papillomavirus (HPV) DNA Vaccine (AMV002) for HPV-Associated Head and Neck Cancer (HNC). Cancer Immunol Immunother (2021) 70(3):743–53. doi: 10.1007/s00262-020-02720-7 PMC10991317 32918586

[92] Aggarwal C Cohen RB Morrow MP Kraynyak KA Sylvester AJ Knoblock DM. Immunotherapy Targeting HPV16/18 Generates Potent Immune Responses in HPV-Associated Head and Neck Cancer. Clin Cancer Res (2019) 25(1):110–24. doi: 10.1158/1078-0432.CCR-18-1763 PMC6320307 30242022

[93] Bommireddy R Munoz LE Kumari A Huang L Fan Y Monterroza L. Tumor Membrane Vesicle Vaccine Augments the Efficacy of Anti-PD1 Antibody in Immune Checkpoint Inhibitor-Resistant Squamous Cell Carcinoma Models of Head and Neck Cancer. Vaccines (Basel) (2020) 8(2):182. doi: 10.3390/vaccines8020182 PMC7348725 32295135

[94] Paolini F Curzio G Cordeiro MN Massa S Mariani L Pimpinelli F. HPV 16 E5 Oncoprotein is Expressed in Early Stage Carcinogenesis and can be a Target of Immunotherapy. Hum Vaccin Immunother (2017) 13(2):291–7. doi: 10.1080/21645515.2017.1264777 PMC5328231 27929754

[95] Ilahi NE Bhatti A. Impact of HPV E5 on Viral Life Cycle *via* EGFR Signaling. Microb Pathog (2020) 139:103923. doi: 10.1016/j.micpath.2019.103923 31836496

[96] Chandra J Dutton JL Li B Woo WP Xu Y Tolley LK. DNA Vaccine Encoding HPV16 Oncogenes E6 and E7 Induces Potent Cell-Mediated and Humoral Immunity Which Protects in Tumor Challenge and Drives E7-Expressing Skin Graft Rejection. J Immunother (2017) 40(2):62–70. doi: 10.1097/CJI.0000000000000156 28166181 PMC5293162

[97] Klinghammer KF Gauler TC Stromberger C Kofla G de Wit M Gollrad J. DURTRERAD: A Phase II Open-Label Study Evaluating Feasibility and Efficacy of Durvalumab (D) and Durvalumab and Tremelimumab (DT) in Combination With Radiotherapy (RT) in non-Resectable Locally Advanced HPV-Negative HNSCC—Results of the Preplanned Feasibility Interim Analysis. J Clin Oncol (2020) 38(15_suppl):6574–4. doi: 10.1200/JCO.2020.38.15_suppl.6574

[98] Taberna M Mena M Tous S Pavón MA Oliva M León X. HPV-Relatedness Definitions for Classifying HPV-Related Oropharyngeal Cancer Patient do Impact on TNM Classification and Patients’ Survival. PloS One (2018) 13(4):e0194107 doi: 10.1371/journal.pone.0194107 29664911 PMC5903634

[99] Chen SW Li SH Shi DB Jiang WM Song M Yang AK. Expression of PD-1/PD-L1 in Head and Neck Squamous Cell Carcinoma and its Clinical Significance. Int J Biol Markers (2019) 34(4):398–405. doi: 10.1177/1724600819884722 31674884

[100] Zandberg DP Algazi AP Jimeno A Good JS Fayette J Bouganim N. Durvalumab for Recurrent or Metastatic Head and Neck Squamous Cell Carcinoma: Results From a Single-Arm, Phase II Study in Patients With ≥25% Tumour Cell PD-L1 Expression Who Have Progressed on Platinum-Based Chemotherapy. Eur J Cancer (2019) 107:142–52. doi: 10.1016/j.ejca.2018.11.015 30576970

[101] Sharma A Seow JJW Dutertre CA Pai R Blériot C Mishra A. Onco-Fetal Reprogramming of Endothelial Cells Drives Immunosuppressive Macrophages in Hepatocellular Carcinoma. Cell (2020) 183(2):377–394.e21. doi: 10.1016/j.cell.2020.08.040 32976798

[102] Sanchez-Martin M Ferrando A. The NOTCH1-MYC Highway Toward T-Cell Acute Lymphoblastic Leukemia. Blood (2017) 129(9):1124–33. doi: 10.1182/blood-2016-09-692582 28115368

[103] Klinakis A Lobry C Abdel-Wahab O Oh P Haeno H Buonamici S. A Novel Tumour-Suppressor Function for the Notch Pathway in Myeloid Leukaemia. Nature (2011) 473(7346):230–3. doi: 10.1038/nature09999 PMC3093658 21562564

[104] Grilli G Hermida-Prado F Álvarez-Fernández M Allonca E Álvarez-González M Astudillo A. Impact of Notch Signaling on the Prognosis of Patients With Head and Neck Squamous Cell Carcinoma. Oral Oncol (2020) 110:105003. doi: 10.1016/j.oraloncology.2020.105003 32932170

[105] Degli Esposti D Sklias A Lima SC Beghelli-de la Forest Divonne S Cahais V Fernandez-Jimenez N. Unique DNA Methylation Signature in HPV-Positive Head and Neck Squamous Cell Carcinomas. Genome Med (2017) 9(1):33. doi: 10.1186/s13073-017-0419-z 28381277 PMC5382363

[106] Ko YH Won HS Sun DS An HJ Jeon EK Kim MS. Human Papillomavirus-Stratified Analysis of the Prognostic Role of miR-21 in Oral Cavity and Oropharyngeal Squamous Cell Carcinoma. Pathol Int (2014) 64(10):499–507. doi: 10.1111/pin.12201 25236707

[107] Ferris RL Blumenschein G Jr. Fayette J Guigay J Colevas AD Licitra L. Nivolumab vs Investigator’s Choice in Recurrent or Metastatic Squamous Cell Carcinoma of the Head and Neck: 2-Year Long-Term Survival Update of CheckMate 141 With Analyses by Tumor PD-L1 Expression. Oral Oncol (2018) 81:45–51. doi: 10.1016/j.oraloncology.2018.04.008 29884413 PMC6563923

[108] Heath BR Michmerhuizen NL Donnelly CR Sansanaphongpricha K Sun D Brenner JC. Head and Neck Cancer Immunotherapy Beyond the Checkpoint Blockade. J Dent Res (2019) 98(10):1073–80. doi: 10.1177/0022034519864112 PMC6704427 31340724

[109] Leach DG Dharmaraj N Piotrowski SL Lopez-Silva TL Lei YL Sikora AG. STINGel: Controlled Release of a Cyclic Dinucleotide for Enhanced Cancer Immunotherapy. Biomaterials (2018) 163:67–75. doi: 10.1016/j.biomaterials.2018.01.035 29454236 PMC5840037

[110] Hato SV Khong A de Vries IJ Lesterhuis WJ. Molecular Pathways: The Immunogenic Effects of Platinum-Based Chemotherapeutics. Clin Cancer Res (2014) 20(11):2831–7. doi: 10.1158/1078-0432.CCR-13-3141 24879823

[111] Deng L Liang H Xu M Yang X Burnette B Arina A. STING-Dependent Cytosolic DNA Sensing Promotes Radiation-Induced Type I Interferon-Dependent Antitumor Immunity in Immunogenic Tumors. Immunity (2014) 41(5):843–52. doi: 10.1016/j.immuni.2014.10.019 PMC5155593 25517616

[112] Pilon-Thomas S Kodumudi KN El-Kenawi AE Russell S Weber AM Luddy K. Neutralization of Tumor Acidity Improves Antitumor Responses to Immunotherapy. Cancer Res (2016) 76(6):1381–90. doi: 10.1158/0008-5472.CAN-15-1743 PMC4829106 26719539

[113] Zhou L Xu N Shibata H Saloura V Uppaluri R. Epigenetic Modulation of Immunotherapy and Implications in Head and Neck Cancer. Cancer Metastasis Rev (2021) 40(1):141–52. doi: 10.1007/s10555-020-09944-0 PMC7897200 33403469

[114] Majumder B Baraneedharan U Thiyagarajan S Radhakrishnan P Narasimhan H Dhandapani M. Predicting Clinical Response to Anticancer Drugs Using an *Ex Vivo* Platform That Captures Tumour Heterogeneity. Nat Commun (2015) 6:6169. doi: 10.1038/ncomms7169 25721094 PMC4351621

[115] Yang J Jiang Q Liu L Peng H Wang Y Li S. Identification of Prognostic Aging-Related Genes Associated With Immunosuppression and Inflammation in Head and Neck Squamous Cell Carcinoma. Aging (Albany NY) (2020) 12(24):25778–804. doi: 10.18632/aging.104199 PMC7803584 33232279

[116] Song D Tian J Han X Li X. A Model of Seven Immune Checkpoint-Related Genes Predicting Overall Survival for Head and Neck Squamous Cell Carcinoma. Eur Arch Otorhinolaryngol (2021) 278(9):3467–77. doi: 10.1007/s00405-020-06540-4 33449165

