# Supplementary material for: An Update on the Immunotherapy for Oropharyngeal Squamous Cell Carcinoma
Source: Front Oncol. 2022 Mar 15;12:800315. doi: 10.3389/fonc.2022.800315 (PMC8965058; doi:10.3389/fonc.2022.800315)
Supplement: Supplementary file 1 [file Table_1.docx]

**Supplementary Table: A comparison of the 7^th^ and 8^th^ edition of UICC/AJCC staging of oropharyngeal squamous cell carcinoma^a^**

|  | **AJCC (7^th^ edition)** | **AJCC (8^th^ edition) ^[37]^** |  |
| --- | --- | --- | --- |
|  |  | **p16-positive OPSCC** | **p16-negative OPSCC** |
| **Primary tumor** | | |  |
| Tx:  T0:  Tis: | Primary tumor cannot be assessed.  No evidence of primary tumor.  Carcinoma in situ. | The same as the 7^th^ edition.  The same as the 7^th^ edition.  -- | The same as the 7^th^ edition.  --  The same as the 7^th^ edition. |
| T1: | Tumor 2 cm or smaller in greatest dimension. | The same as the 7^th^ edition. | The same as the 7^th^ edition. |
| T2: | Tumor larger than 2 cm but not larger than 4 cm in greatest dimension. | The same as the 7^th^ edition. | The same as the 7^th^ edition. |
| T3: | Tumor larger than 4 cm in greatest dimension or extension to lingual surface of epiglottis. | The same as the 7^th^ edition. | The same as the 7^th^ edition. |
| T4:  T4a:  T4b: | Moderately advanced or very advanced local disease.  Moderately advanced local disease; tumor invades the larynx, extrinsic muscle of tongue, medial pterygoid, hard palate, or mandible^b^.  Very advanced local disease; tumor invades lateral pterygoid muscle, pterygoid plates, lateral nasopharynx, or skull base or encases carotid artery. | The same as the 7^th^ edition.  T4b category has been removed from p16-positive OPSCC. | The same as the 7^th^ edition.  The same as the 7^th^ edition. |
| **Regional lymph node status** | | |  |
| Nx:  N0: | Regional lymph node cannot be ssessed.  No regional lymph node metastasis. | The same as the 7^th^ edition.  The same as the 7^th^ edition. | The same as the 7^th^ edition.  The same as the 7^th^ edition. |
| N1: | Metastasis to a single ipsilateral lymph node (≤3 cm). | **cN:** One or more ipsilateral lymph nodes, none larger than 6 cm.  **pN:** Metastasis in 4 or fewer lymph nodes. | **cN/pN:** New introduction of ENE-negative, based on the 7^th^ edition. |
| N2 | Metastasis in a single ipsilateral lymph node larger than 3 cm but not larger than 6 cm in greatest dimension; or metastases in multiple ipsilateral lymph nodes, none larger than 6 cm in greatest dimension; or metastasis in bilateral or contralateral lymph nodes, none larger than 6 cm in greatest dimension. | **cN:** Contralateral or bilateral lymph nodes, none larger than 6 cm.  **pN:** Metastasis in more than 4 lymph nodes. | New introduction of ENE, based on the 7^th^ edition. |
| N2a: | Metastasis in a single ipsilateral lymph node larger than 3 cm but not larger than 6 cm in greatest dimension. | -- | **cN:** New introduction of ENE-negative, based on the 7^th^ edition.  **pN:** New introduction of ENE-negative,based on the 7^th^ edition; Metastasis in a single ipsilateral lymph node 3 cm or less in greatest dimension and ENE-positive. |
| N2b: | Metastasis to multiple ipsilateral lymph nodes (≤6 cm). | -- | **cN/pN:** New introduction of ENE-negative, based on the 7^th^ edition. |
| N2c: | Metastasis to bilateral or contralateral lymph nodes (≤6 cm). | -- | **cN/pN:** New introduction of ENE-negative, based on the 7^th^ edition. |
| N3: | Metastasis to any lymph node (>6 cm). | **cN:** Lymph node(s) larger than 6 cm.  **pN: --** | **cN3a/pN3a:** New introduction of ENE-negative, based on the 7^th^ edition.  **cN3b:** Metastasis in any node(s) and clinically overt ENE-positive.  **pN3b:** The same as the 8^th^ edition unless a single small node (**pN2a**). |
| **Distant metastasis** | | |  |
| Mx: | Cannot be assessed. | The same as the 7^th^ edition. | The same as the 7^th^ edition. |
| M0: | No distant metastasis. | The same as the 7^th^ edition. | The same as the 7^th^ edition. |
| M1: | Distant metastasis. | The same as the 7^th^ edition. | The same as the 7^th^ edition. |

Abbreviations: UICC/AJCC, the Union for International Cancer Control and American Joint Committee on Cancer; ENE, extranodal extension; OPSCC, oropharyngeal squamous cell carcinoma.

^a^ The original source for this material is the AJCC Cancer Staging Manual, Eighth Edition (2017) published by Springer Science and Business Media LLC (springer.com) (Amin MB, Edge SB, Greene FL, et al, eds. AJCC Cancer Staging Manual. 8th ed. New York: Springer; 2017).

^b^ Mucosal extension to lingual surface of epiglottis from primary tumors of the base of the tongue and vallecula does not constitute invasion of the larynx.
